# Supplementary material for: Alterations in germinal center formation and B cell activation during severe Orientia tsutsugamushi infection in mice
Source: PLoS Negl Trop Dis. 2023 May 5;17(5):e0011090. doi: 10.1371/journal.pntd.0011090 (PMC10191367; doi:10.1371/journal.pntd.0011090)
Supplement: S1 Table — (PDF) [file pntd.0011090.s001.pdf]

| Gene Target | Forward 5’-3’ | Reverse 5’-3 prime |
| --- | --- | --- |
| *CCR7* | TGTACGAGTCGGTGTGCTTC | GGTAGGTATCCGTCATGGTCTTG |
| *CD40* | TGTCATCTGTGAAAAGGTGGTC | ACTGGAGCAGCGGTGTTATG |
| *CD86* | TGTTTCCGTGGAGACGCAAG | TTGAGCCTTTGTAAATGGGCA |
| *CXCR5* | ATGAACTACCCACTAACCCTGG | TGTAGGGGAATCTCCGTGCT |
| *ICOS-L* | TAAAGTGTCCCTGTTTTGTGTCC | ATTGCACCGACTTCAGTCTCT |
| *IL-4* | GGTCTCAACCCCCAGCTAGT | GCCGATGATCTCTCTCAAGTGAT |
| *IL-6* | TAGTCCTTCCTACCCCAATTTCC | TTGGTCCTTAGCCACTCCTTC |
| *IL-21* | GGACCCTTGTCTGTCTGGTAG | TGTGGAGCTGATAGAAGTTCAGG |
| *IFN-γ* | ATGAACGCTACACACTGCATC | CCATCCTTTTGCCAGTTCCTC |
| *SLAMF1* | CAGAAATCAGGGCCTCAAGAG | CACTGGCATAAACTGTGGTGG |
| GAPDH | TGGAAAGCTGTGGCGTGAT | TGCTTCACCACCTTCTTGAT |
| *O. tsutsugamushi* 47-kDa | AACTGATTTTATTCAAACTAATGCTGCT | TATGCCTGAGTAAGATACTGTAATGGA |
