# Supplementary figures and images for: Alterations in germinal center formation and B cell activation during severe Orientia tsutsugamushi infection in mice
Source: PLoS Negl Trop Dis. 2023 May 5;17(5):e0011090. doi: 10.1371/journal.pntd.0011090 (PMC10191367; doi:10.1371/journal.pntd.0011090)

**A**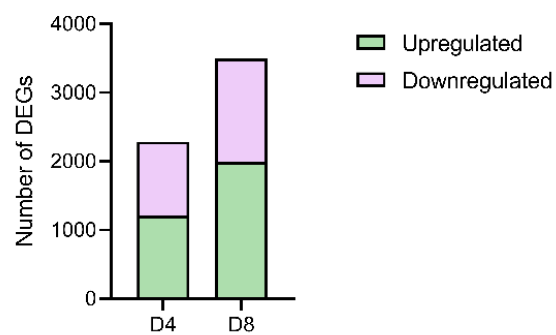**B****D4 vs Mock**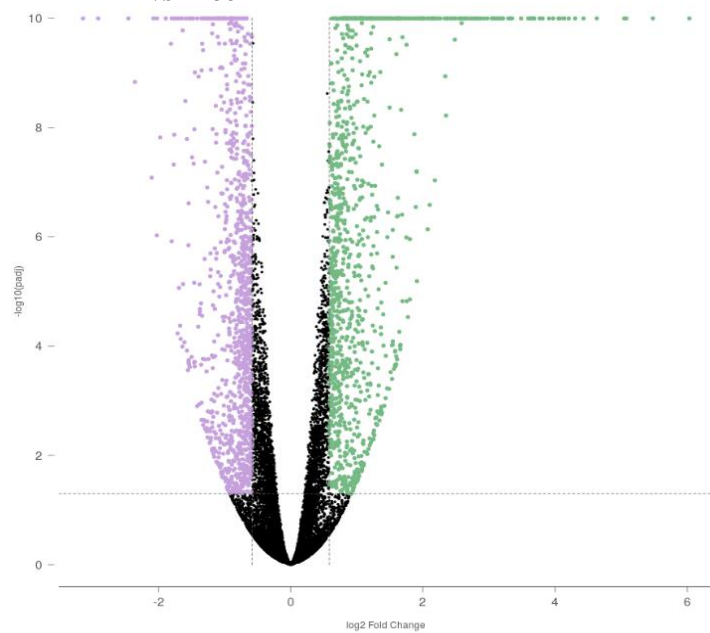**C****D8 vs Mock**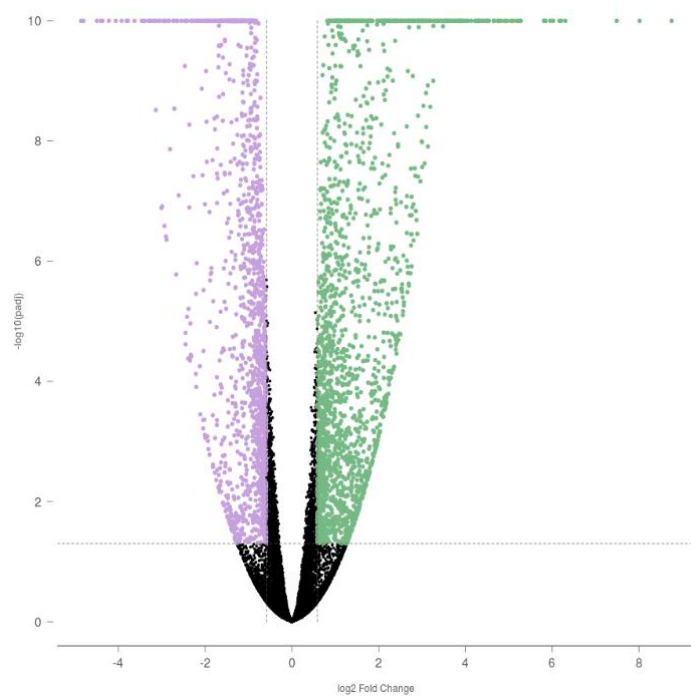

Supplement: S1 Fig — Mice were infected, as described in Fig 1; splenic B cells were purified from mock samples (n = 2) and at D4 (n = 3) and D8 (n = 3) for RNAseq analyses. Data were normalized via Relative Log Expression (RLE) using DESeq2 R library. Differentially expressed genes for from D4 and D8 samples identified by ROSALIND analysis, relative to mock, are shown in the A) bar graph and B-C) volcano plots. (PDF) [file pntd.0011090.s002.pdf]

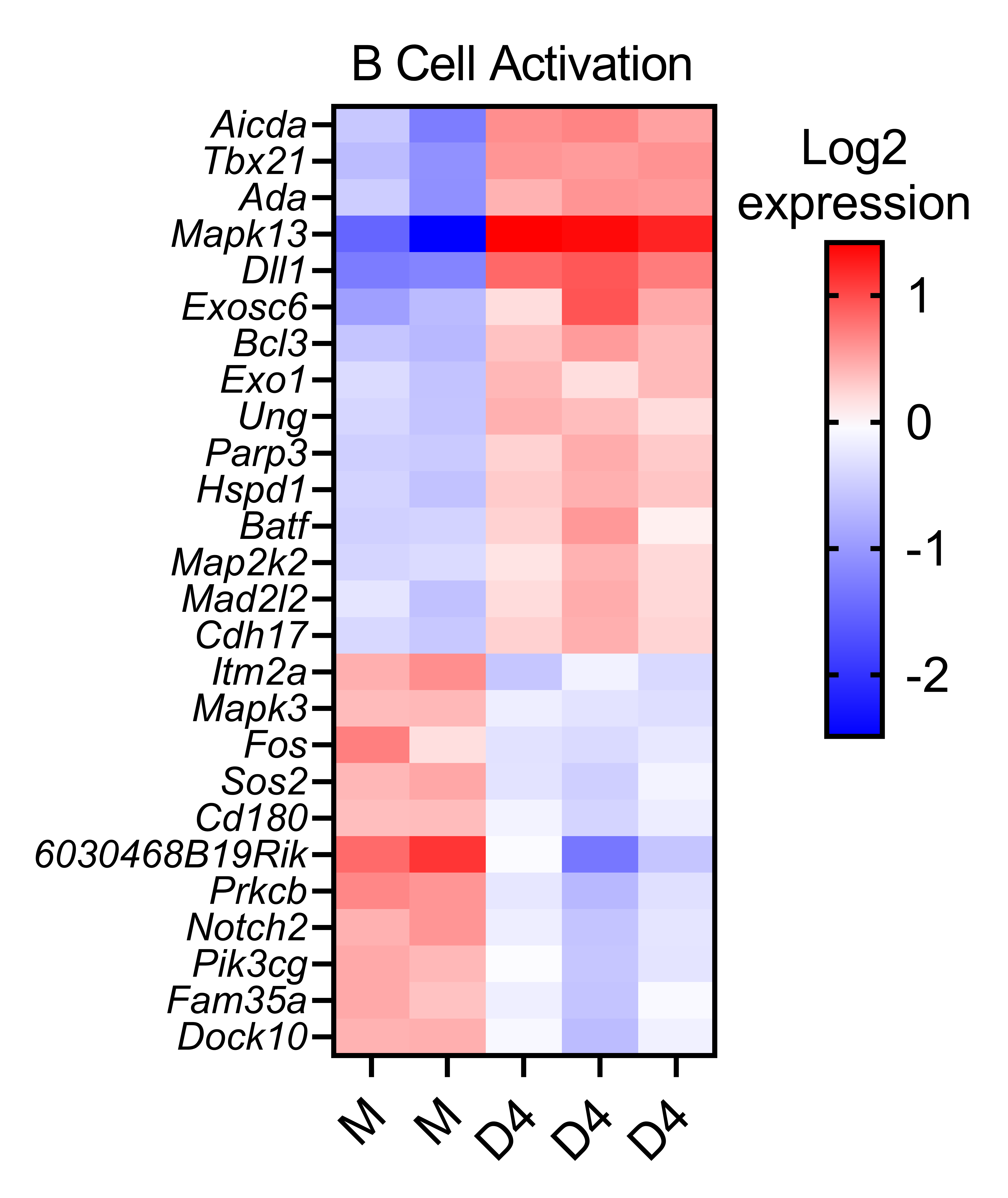

Supplement: S2 Fig — Mice were infected, as described in Fig 1; splenic B cells were purified from mock samples (n = 2) and at D4 (n = 3) and D8 (n = 3) for RNAseq analyses. Data were normalized via Relative Log Expression (RLE) using DESeq2 R library. Differentially expressed genes for B cell activation (PANTHER and Gene Ontology Biological Process databases) from mock and D4 samples were identified by ROSALIND analysis and listed in the heatmap as subtracted normalized Log2 expression values. (TIF) [file pntd.0011090.s003.tif]

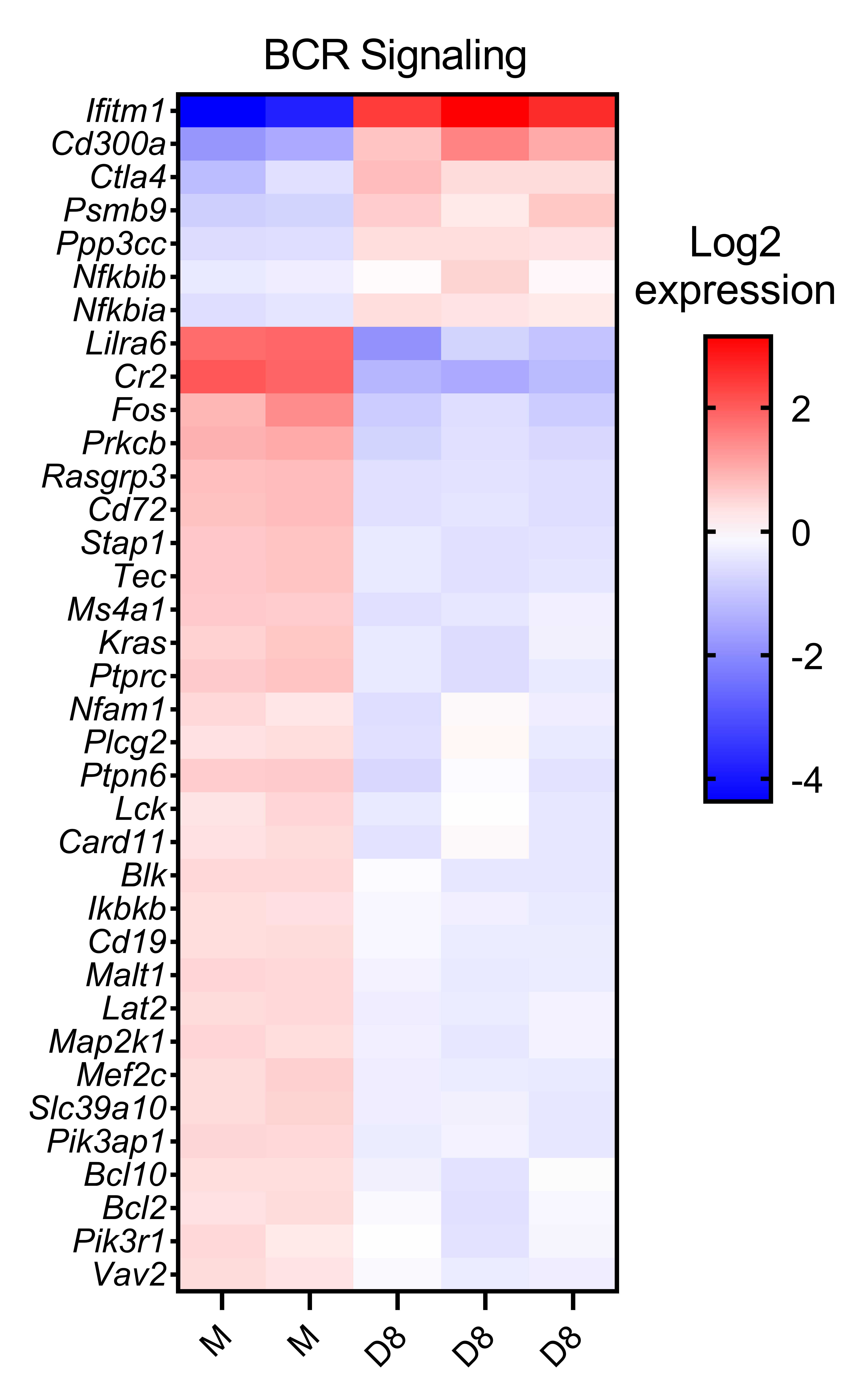

Supplement: S3 Fig — Mice were infected, as described in Fig 1; splenic B cells were purified from mock samples (n = 2) and at D4 (n = 3) and D8 (n = 3) for RNAseq analyses. Data were normalized via Relative Log Expression (RLE) using DESeq2 R library. Differentially expressed genes for BCR signaling (KEGG and Gene Ontology Biological Process databases) from mock and D8 samples were identified by ROSALIND analysis and listed in the heatmap as subtracted normalized Log2 expression values. (TIF) [file pntd.0011090.s004.tif]
